# Diagnostic Value and Operational Recommendations for Late Iodine Enhancement and ECV Quantification in Single-Energy Computed Tomography: A Narrative Review

**DOI:** 10.3390/diagnostics16162547

**Published:** 2026-08-12

**Authors:** Simone Steffani, Mariagrazia Piscione, Dario Gaudio, Giorgia Meghnagi, Viviana Montella, Francesco Fiorini, Andrea Micillo, Corrado Tagliati, Luigi Asmundo, Guglielmo Manenti, Marcello Chiocchi, Mario Laudazi

**Affiliations:** 1 Department of Biomedicine and Prevention, University of Rome Tor Vergata, Via Montpellier 1, 00133 Rome, Italy; 2 Department of Cardiology, SS. Annunziata Hospital, ASL2, 66100 Chieti, Italy; 3 Fondazione Policlinico Campus Bio-Medico, University of Rome, Via Alvaro del Portillo 200, 00128 Rome, Italy; 4 Diagnostic Imaging Unit, Casa di Cura Villa Delle Querce, 00040 Nemi, Italy; 5 AST Ancona, Ospedale di Comunità Maria Montessori di Chiaravalle, Via Fratelli Rosselli 176, 60033 Chiaravalle, Italy; 6 Department of Radiology, Ospedale Niguarda Ca’ Granda, Piazza Ospedale Maggiore, 20162 Milan, Italy; 7 Diagnostic Imaging Department, AOU Policlinico Tor Vergata, 00133 Rome, Italy

**Keywords:** single-energy computed tomography, late iodine enhancement, extracellular volume, cardiac computed tomography, myocardial tissue characterization

## Abstract

While traditionally focused on coronary anatomy, cardiac computed tomography now enables non-invasive myocardial tissue characterization. By evaluating late iodine enhancement (LIE) and extracellular volume (ECV), single-energy CT (SECT) provides a valuable alternative to cardiac magnetic resonance for assessing ischemic and non-ischemic pathologies. However, clinical implementation of SECT faces technical challenges, primarily the low contrast-to-noise ratio (CNR) of iodine and the reliance on image subtraction for ECV quantification, both of which increase radiation exposure and susceptibility to spatial misregistration. To address these issues, protocol optimization is essential. Evidence-based recommendations include using low tube voltages to shift the X-ray spectrum closer to the iodine K-edge, paired with high reference tube currents. Additionally, delayed acquisition timing should be tailored to specific pathological targets to account for differences in contrast kinetics, and advanced iterative or deep learning image reconstructions should be implemented to mitigate noise. Optimized SECT demonstrates diagnostic and prognostic utility in conditions like acute myocardial infarction, hypertrophic cardiomyopathy, cardiac amyloidosis, and left ventricular thrombus detection. While spectral imaging represents the future, optimizing SECT through technical adjustments and standardized training is crucial for integrating myocardial characterization into routine workflows.

## 1. Introduction

Cardiac computed tomography (CT) has evolved from an anatomical tool for coronary assessment into a modality capable of myocardial tissue characterization [1,2,3]. This progress is based on the pharmacokinetics of iodinated contrast media, which distribute into the extracellular space in a manner similar to gadolinium-based agents used in magnetic resonance imaging [1,2,4,5]. By identifying regions of late iodine enhancement (LIE) and quantifying the extracellular volume (ECV) fraction, CT provides a non-invasive assessment of myocardial alterations, such as fibrosis and infiltrative processes [1,6,7,8]. These capabilities allow for the evaluation of both ischemic and non-ischemic pathologies within a single comprehensive evaluation [2,3,9]. While Cardiac Magnetic Resonance (CMR) with Late Gadolinium Enhancement (LGE) remains the reference standard, its widespread adoption is hindered by extended acquisition times and patient-specific contraindications [1,2,4,10,11]. Consequently, SECT has emerged as a valuable alternative [1,2,4,6,12]. However, SECT faces technical challenges that can affect its diagnostic reliability [4,5]. A primary limitation is the lower contrast-to-noise ratio (CNR) of iodine compared to gadolinium, which may limit the detection of subtle or millimetric myocardial defect [1,5,13,14,15]. Furthermore, the quantification of ECV in SECT relies exclusively on image subtraction techniques that are susceptible to spatial misregistration and require additional baseline scans, which incrementally increase cumulative radiation exposure [1,5,6,16,17,18]. Additionally, the qualitative interpretation of these images is dependent on operator experience, highlighting a need for procedural standardization [1,2,3,15]. Although the field is moving towards spectral imaging (such as dual-energy (DECT) and photon-counting CT (PCD-CT)) to maximize CNR, the global installed base currently relies heavily on SECT scanners [1,8,11,12,18,19]. Therefore, maximizing their diagnostic potential through targeted technical optimization remains important [2,6,11,13,14,18]. While previous reviews have addressed the general diagnostic accuracy of CT, they leave unresolved the specific operational protocols required to optimize SECT in standard clinical practice, particularly regarding the necessary trade-offs between image noise reduction, radiation dose, and spatial resolution. This narrative review aims to evaluate the diagnostic and prognostic value of LIE and ECV quantification, specifically focusing on the SECT modality across various cardiac conditions. The objective is to provide evidence-based operational recommendations for protocol optimization to address technical limitations. By synthesizing current evidence, this work seeks to offer a framework for integrating myocardial tissue characterization into routine diagnostic workflows.

## 2. Methods

A bibliographic search was performed in April 2026 in the Scopus (Elsevier, Amsterdam, the Netherlands), MEDLINE (US National Library of Medicine, Bethesda, MD, USA), and Web of Science (Thomson Reuters, Toronto, ON, Canada) databases. Combinations of the following descriptors were used according to a free-text protocol: “single-energy computed tomography”, “dual-energy computed tomography”, “photon counting”, “cardiac computed tomography”, “late iodine enhancement”, “delayed enhancement”, “extracellular volume”, “myocardial tissue characterization”, “ischemic heart disease”, “non-ischemic heart disease”. The literature search was restricted to articles in English published from the year 2000. Emphasis was focused on peer-reviewed original research, reviews, and meta-analyses explicitly reporting on SECT acquisition protocols, contrast media kinetics, and diagnostic performance for myocardial tissue characterization. Conference abstracts, case reports, and commentaries were excluded. Consistent with the design of a purely narrative and educational review, a formal systematic screening process was not conducted. Instead, articles were purposively selected by the authors based on their clinical relevance, scientific merit, and contribution to the technological advancement in SECT imaging. Consequently, formal systematic counts, flowcharts, and risk-of-bias assessments were not applicable. Results were narratively reported.

## 3. Pathophysiological Principles of Late Iodine Enhancement

### 3.1. Iodine Kinetics

**Figure 1:**
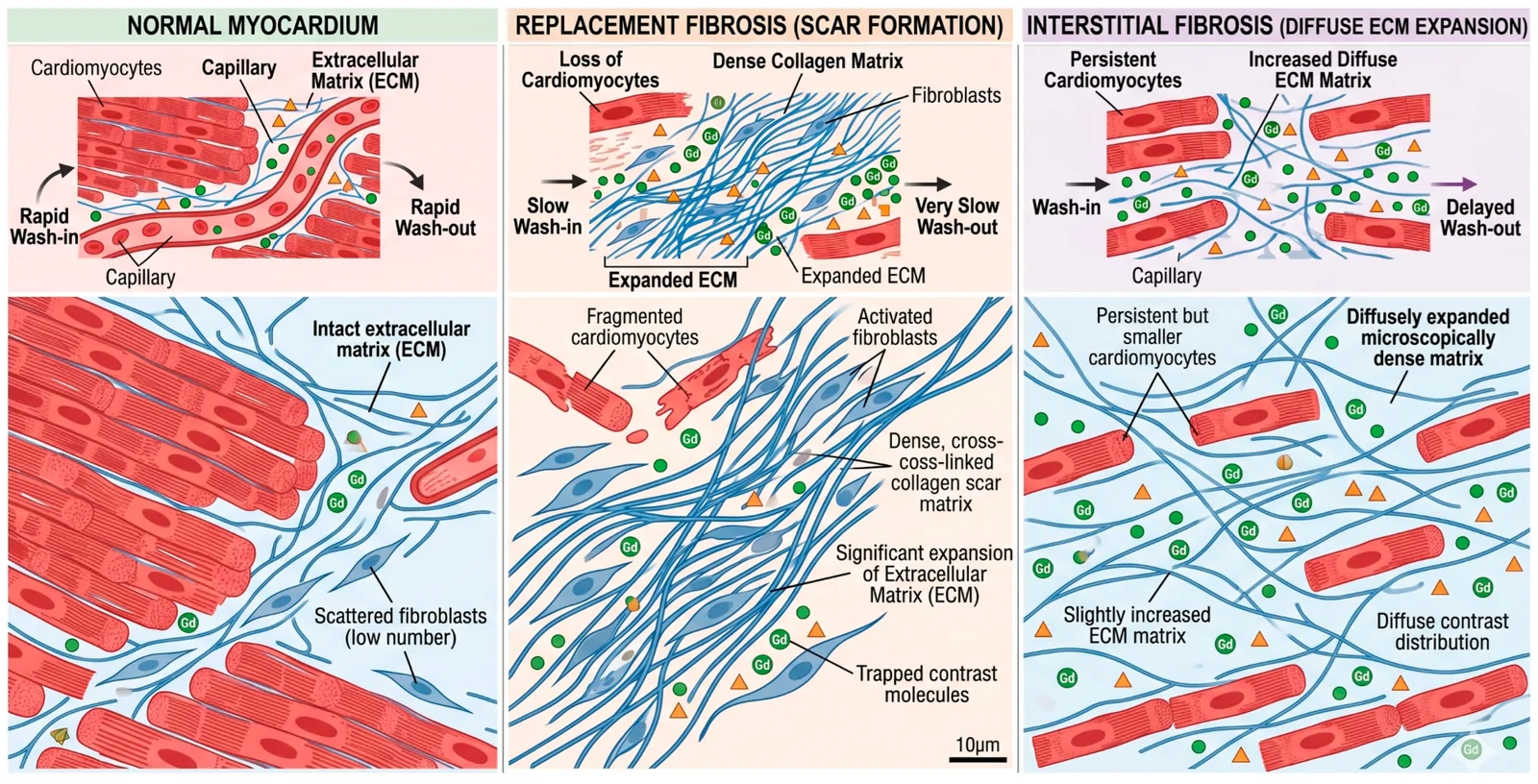
Schematic representation of myocardial histology and Gadolinium/Iodine pharmacokinetics. Normal myocardium allows rapid contrast wash-in and wash-out. Replacement fibrosis traps contrast in dense collagen scars, causing delayed wash-out and focal late enhancement. Diffuse interstitial fibrosis features an expanded extracellular matrix with normal wash-in but delayed wash-out. Both agents exhibit parallel strictly extracellular kinetics. Gd = Gadolinium; Orange triangle = Iodine.

The evaluation of LIE in cardiac CT relies on the specific pharmacokinetics of iodinated contrast media, which distribute rapidly and passively from the intravascular compartment into the extracellular space down a concentration gradient, without penetrating the intact intracellular space [2,6]. In healthy myocardial tissue, the tightly packed myocytes restrict the extracellular space, resulting in a rapid wash-in and subsequent wash-out of the contrast agent [1,2,20,21]. Conversely, in pathological states (acute necrosis, chronic fibrotic scarring, or extracellular deposition), the extracellular volume appears to be expanded due to myocyte membrane rupture or collagen accumulation [1,2,4,6,7]. This expansion, combined with reduced capillary density in fibrotic scars, has been shown to lead to altered contrast kinetics characterized by delayed wash-in and prolonged wash-out [2,9,21,22,23] (Figure 1). Consequently, at a LIE imaging timepoint, the iodinated contrast medium is retained in the damaged myocardial regions, establishing a concentration gradient between the scarred tissue and the remote, healthy myocardium [1,2,4,6].

### 3.2. Iodine–Gadolinium Parallel

Iodinated contrast agents utilized in CT share notable pharmacokinetic similarities with gadolinium-based chelates, as both function as extracellular and extravascular contrast agents [1,2,6]. Just as gadolinium retention shortens the T1 relaxation time to produce hyperintense LGE signals, the regional accumulation of iodine proportionally increases the tissue attenuation measured in Hounsfield Units (HU) [2,5,7]. Fibrotic or infarcted myocardial regions typically present as hyperdense areas on SECT-LIE scans [5,7,18,24]. The primary physical discrepancy lies in the X-ray attenuation of iodine, which yields a more subtle signal difference compared to gadolinium’s stark hyperintensity, explaining the inherently lower CNR of SECT [9,14,20,23]. Despite this limitation, experimental models have corroborated the biological parallel, demonstrating a strong spatial correlation between histopathological alterations and the regions of LIE identified on CT [1,20,24]. These findings confirm that the iodine–gadolinium parallel provides a reliable biological foundation for translating LGE principles to SECT-based tissue characterization [1,2,17,23,25].

## 4. Optimization of SECT Acquisition Protocol for LIE

To achieve high-quality LIE imaging and ensure diagnostic reproducibility, several technical parameters should be optimized [4,19]. Table 1 provides a comprehensive overview of the recommended acquisition protocol, which is discussed in detail in the following subsections.

### 4.1. Contrast Media Administration

To partially compensate for the inherently lower CNR of SECT compared to late gadolinium enhancement magnetic resonance imaging (LGE-MRI), the administration protocol of iodinated contrast media must be carefully tailored to optimize iodine delivery to the interstitial space [1,6,17]. It is recommended to utilize a high-concentration contrast agent (e.g., 350–400 mg I/mL) to increase overall myocardial attenuation [1,5,24,26,27,28]. Historically, contrast administration schemes have often been extrapolated from early, small-scale validation studies of acute myocardial infarction, such as the protocol of 1.5 mL/kg proposed by Jacquier et al. [21]. However, iodinated contrast media kinetics differ substantially across different myocardial substrates, meaning that a single standardized dose is not universally appropriate [1,16,17,20,21,23,28,33].

To ensure adequate CNR for LIE focal scar visualization, clinical protocols in the literature frequently report using weight-dependent contrast volumes typically ranging from 1.4 to 1.8 mL/kg of high-concentration contrast agent [1,6,10,29]. Rather than representing a prospective mathematical threshold of “optimal enhancement”, this weight-based range serves as a pragmatic clinical standard to guarantee acceptable lesion delineation across varying patient sizes, particularly in obese patients [1,2,6,30]. This standard weight-based protocol remains highly beneficial for the visual detection of subtle, subendocardial focal scars because lower doses can hinder visual scar detection [3,12,17,30,42]. A commonly reported strategy to achieve sufficient interstitial accumulation of contrast material without compromising the initial coronary CT angiography (CCTA) phase is a split-bolus or triphasic injection approach [1,4,28,48]. In these protocols, an initial bolus of undiluted contrast is administered for the angiographic phase, followed by a slower contrast infusion or a contrast-saline mixture (e.g., 40–50 mL of a 50% contrast-saline mixture administered at 3–4 mL/s), and concluding with a saline flush (e.g., 20–40 mL of pure saline) to minimize perivenous streak and beam-hardening artifacts [3,4,11,15,19,28].

### 4.2. Acquisition Timing (Delay Time)

The timing for acquiring the LIE scan is governed by the equilibrium kinetics of the contrast agent [1,2,6,10,35]. For example, in the setting of acute, reperfused myocardial infarction, Jacquier et al. demonstrated that a 5 min delay yields a significantly higher SNR and better subjective image quality than a 10 min acquisition, as it captures peak hyperenhancement while avoiding excessive renal contrast clearance [21]. In chronic myocardial scars, myocardial tissue remodeling is characterized by stable collagenous replacement, resulting in slower dynamic wash-in and wash-out kinetics; here, Hamdy et al. showed that contrast kinetics are more stable, and delayed acquisitions at 5 to 7 min provide good scar-to-myocardium delineation and highly consistent ECV measurements [2,14,20,35]. In diffuse infiltrative disease such as cardiac amyloidosis, the massive expansion of the extracellular space by amyloid fibrils leads to a progressive, time-dependent accumulation of iodinated contrast from the blood pool into the interstitium; consequently, Treibel et al. demonstrated that a shorter delay of 5 min is superior to 15 min, as the latter suffers from severe signal degradation and lower CNR [1,6,33,34]. For acute inflammatory states such as active myocarditis, longer delays of approximately 10 min are suggested to allow adequate blood-pool clearance and differentiate subepicardial or mid-wall inflammatory patterns [6,18]. In other non-ischemic conditions, such as hypertrophic cardiomyopathy (HCM), clinical validation protocols suggest a delay of 7 min to ensure adequate washout of the normal myocardium [10,26,32]. Similarly, in non-ischemic dilated cardiomyopathy, SECT protocols have successfully implemented delayed scan times ranging from 6 to 10 min to visualize myocardial delayed enhancement [11,15,31]. Thus, while a 5–10 min range serves as a general guide, scan timing should be tailored to the specific pathophysiological target to maximize diagnostic accuracy.

### 4.3. X-Ray Tube Parameters (kVp and mAs)

The modulation of X-ray tube parameters represents a relevant approach in optimizing SECT for LIE, particularly through the use of a low tube voltage [1,5]. Because the K-edge of iodine is 33.2 keV, reducing the tube voltage to 80 kVp significantly increases iodine attenuation because the mean energy of the X-ray spectrum shifts closer to the iodine K-edge compared to standard 120 kVp protocols [3,5,36,37]. This shift maximizes the photoelectric effect, enhancing the contrast between scarred and healthy tissue [3]. However, the reduction in tube voltage intrinsically increases image noise, which can degrade diagnostic confidence, especially in obese patients [1,3,30,36]. To mitigate this limitation, clinical studies by Bettencourt et al. and Palmisano et al. support a weight- or BMI-adapted tube voltage strategy (80 kVp for patients with BMI < 30 kg/m^2^ and 100 kVp for patients with BMI ≥ 30 kg/m^2^), which successfully prevents ‘photon starvation’ while preserving the elevated iodine attenuation necessary for scar identification [3,4,14,30]. It is also recommended to employ automatic exposure control (AEC) systems programmed with a high reference tube current-time product (e.g., a reference of 500–600 mAs) to compensate for the lower kilovoltage [11,13,22,35,36,39].

### 4.4. Reconstruction Algorithms and Noise Reduction

Standard filtered back projection (FBP) algorithms are often inadequate for low-voltage SECT LIE scans due to unacceptably high image noise [40]. The implementation of advanced iterative reconstruction (IR) and knowledge-based iterative model reconstruction (IMR) techniques has been shown to improve the objective and subjective quality of the images [40]. Tanabe et al. evaluated 35 patients using a 256-slice CT scanner, demonstrating that the median SNR progressively increased from 2.1 using FBP, to 2.9 with hybrid IR, and up to 6.1 with IMR [40]. Concurrently, the median CNR improved from 1.7 (FBP) to 4.7 (IMR) [40]. This noise reduction directly affected diagnostic performance, as the sensitivity for detecting myocardial infarction increased from 56% with FBP to 80% with IMR [40]. Furthermore, recent data suggest that Deep Learning Image Reconstruction (DLIR) may provide superior noise suppression and spatial resolution preservation compared to conventional IR [11,13]. In a study by Aoki et al. involving 50 patients evaluated with a 256-row CT at 70 kVp, the application of DLIR yielded a high CNR of 4.3 ± 1.2, compared to 2.3 ± 0.7 for standard IR [11]. The diagnostic performance using DLIR was robust, achieving a per-patient sensitivity of 94%, specificity of 100%, and an overall accuracy of 96% when compared to MRI as the reference standard [11]. Finally, the integration of post hoc deep learning filters is supported by Nishii et al., who demonstrated that a residual dense network denoising filter trained on averaged MDE images significantly improves visual assessment, elevating sensitivity from 77.9% to 93.1% compared to original single-shot images [13]. Additional noise reduction can be achieved through spatial frequency filtration and image averaging, where multiple consecutive heartbeats are acquired and co-registered to smooth the background noise [5,6,13,35,43].

### 4.5. Image Slice Thickness and Voxel Optimization

A technical trade-off exists in single-energy computed tomography (SECT) between voxel-level signal-to-noise ratio (SNR) and spatial resolution along the z-axis [8,14,20,45]. While high spatial resolution (typically 0.5 to 0.625 mm slice thickness) is standard for coronary artery evaluation, the qualitative and quantitative assessment of LIE on SECT has historically favored thicker reconstructed image slices (e.g., 3 to 10 mm) to suppress background image noise and artificially boost the visual CNR [5,14,16,17,44]. Indeed, reconstructing thicker slices significantly improves the signal-to-noise ratio per voxel, thereby suppressing quantum noise and improving contrast resolution without necessitating an increase in the patient’s radiation dose [5,8,16,17,20,44]. However, reconstructing excessively thick slices significantly aggravates z-axis partial-volume averaging [8,20,49]. This effect blurs the anatomical interfaces between the myocardium, the blood pool, and epicardial fat, directly compromising the detection of subtle or millimetric subendocardial scars [8,9,30].

This partial-volume averaging also hinders the precise grading of myocardial scar transmurality (i.e., distinguishing ≤ 50% vs. >50% of wall thickness), which serves as the decisive clinical threshold for determining myocardial viability [4,9,22,45]. To resolve this technical limitation within the SECT modality, current protocols recommend a hybrid reconstruction and visualization strategy [5,17]. Axial images should be reconstructed with a thin slice thickness (typically 1.0 to 1.5 mm, or 0.75 mm) to preserve spatial resolution, minimize partial-volume effects, and allow accurate multiplanar reformations [10,17,24,39]. Thicker short-axis reformations (e.g., 5 to 10 mm) in average intensity projection with narrow window settings (typically W300, L150) are utilized to facilitate rapid visual screening, but they should always be cross-referenced with the thin-slice datasets to prevent the omission of subtle subendocardial lesions [2,10,30,35,44].

## 5. Extracellular Volume (ECV) Quantification with SECT

**Figure 2:**
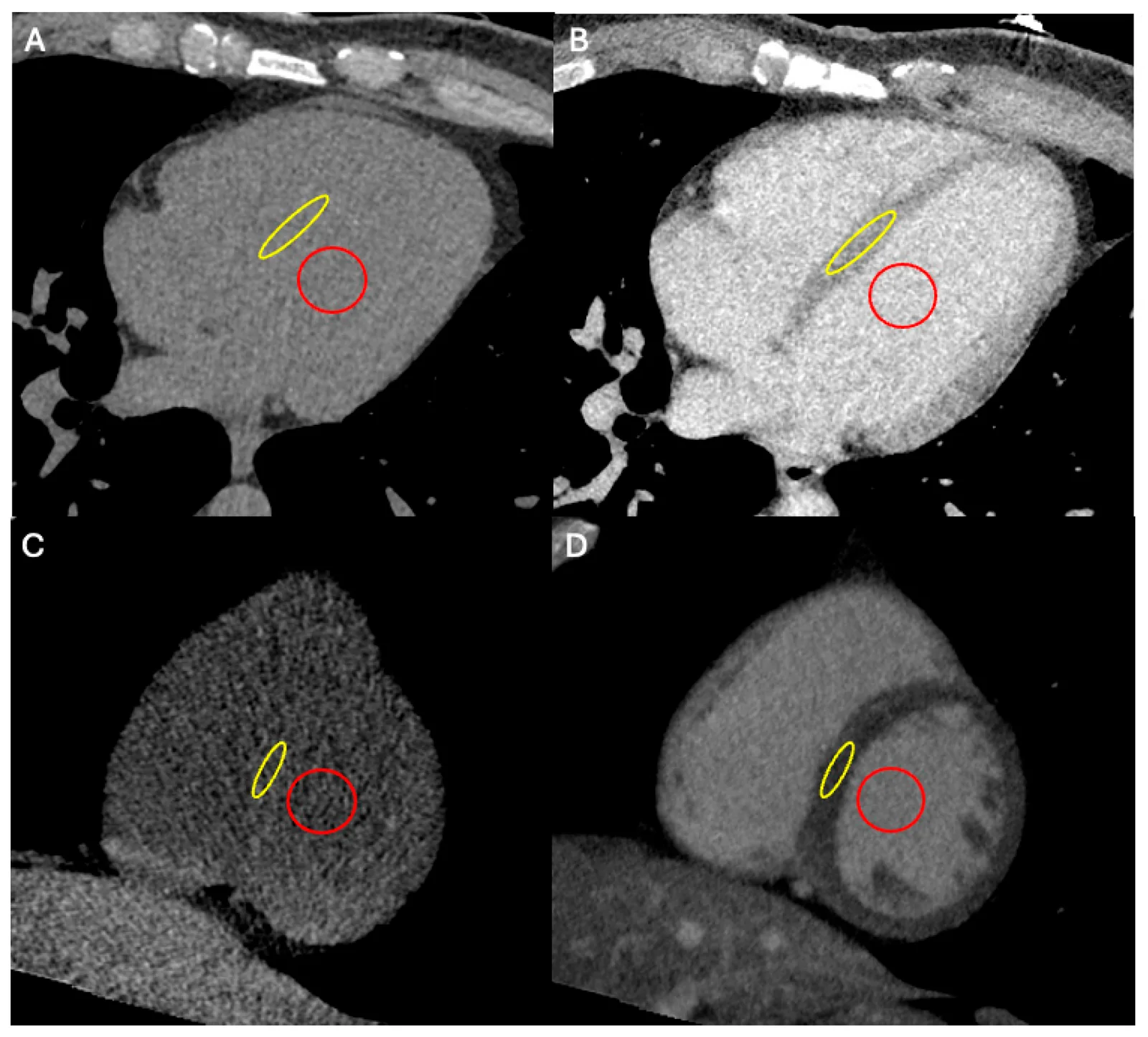
ECV measurement using the subtraction method in single-energy computed tomography (SECT). Regions of interest (ROIs) were initially drawn in the mid-ventricular septum (yellow ROI) and in the left ventricular blood pool (red ROI) on the late iodine enhancement (LIE) post-contrast scans in four-chamber (**B**) and mid-ventricular short-axis (**D**) views. To ensure constant ROI dimensions and precise anatomical location, these ROIs were subsequently copied onto the exact same locations on the co-registered non-enhanced baseline scans (**A**,**C**, respectively). The use of automatic, three-dimensional non-rigid image registration is recommended to electronically align the myocardial borders, thereby mitigating spatial misregistration artifacts.

Computed tomography allows for the quantification of the ECV fraction to comprehensively assess diffuse interstitial fibrosis or infiltrative processes [16,27,31,50]. The ECV is a physiological metric that measures the relative expansion of the cardiac extracellular matrix (representing the interstitial and vascular spaces), while excluding the intracellular compartment [2,5,6,42,51,52,53]. In healthy cohorts, CT-ECV estimates exhibit substantial variation in the literature, ranging from 21.6% to 35.1%, with a pooled meta-analysis estimate of 27.6% [16]. This heterogeneity is driven by technical and protocol differences, including single-energy subtraction versus dual-energy or spectral acquisitions, variable scan delays, and region of interest selection [17,27,48]. Furthermore, measurements are influenced by CT reconstruction parameters, such as slice thickness and kernel selection, as well as the use of synthetic versus directly measured hematocrit [8,16,48,54]. A pathological increase in ECV, on the other hand, serves as a non-invasive biomarker for diffuse collagen deposition, edema, or amyloid infiltration [17,27,31]. Unlike DECT and PCD-CT technologies, which can calculate ECV from a single LIE scan using iodine density maps, SECT relies on a subtraction method [1,5,50]. This technique requires the acquisition of two distinct ECG-gated scans: a non-enhanced baseline scan (which must be acquired at the exact same tube voltage as the LIE scan) and a LIE scan [1,3,5,6,16]. The global or regional distribution of the contrast medium is calculated by measuring the attenuation (in Hounsfield Units, HU) within specific regions of interest (ROIs) drawn in the left ventricular myocardium and the blood pool. By subtracting the pre-contrast attenuation from the delayed post-contrast attenuation, the ΔHU is obtained [1,2,5,38]. The SECT-ECV is subsequently derived using the formula: ECV = (1 − Hematocrit) × (ΔHUmyocardium/ΔHUblood-pool) [1,2,5,38]. To accurately normalize the contrast concentration to the patient’s plasma volume, a venous blood draw for hematocrit measurement on the same day as the exam is required [1,5,6]. Regarding the planes for analysis, ROIs are typically traced on multiplanar reformations, primarily using the short-axis and four-chamber planes [11,27,30,42]. Mid-level four-chamber and mid-level short-axis images, placing a single ROI on the interventricular septum is a common and practical approach that correlates well with the overall degree of diffuse fibrosis, acting as a reliable marker for the entire ventricle [16,27,55] (Figure 2). However, to obtain a better and more precise quantification, it is recommended to perform a global measurement by tracing 16 or 17 ROIs according to the standard American Heart Association segmentation model [10,16,27,32,35,38,48].

To ensure precision, it is recommended to draw ROIs that encompass the maximum possible area of the myocardial septum while strictly avoiding the endocardial and epicardial borders to prevent partial volume averaging effects with the blood pool or epicardial fat [8,16,27,56,57]. Although a universally standardized ROI size for ECV calculation is lacking, the literature demonstrates significant variability. For myocardial tissue assessment, studies report utilizing ROIs ranging from as small as 10 to 30 mm^2^ up to larger areas exceeding 50 or 100 mm^2^ [9,11,19,29,35,42,58]. Conversely, for the left ventricular blood pool, substantially larger ROIs (typically ranging from 50 mm^2^ to 200 mm^2^) are often employed to acquire a representative attenuation sample [13,27,38,42,56]. Regardless of the absolute dimensions chosen, it is essential to keep the size and location of the ROIs strictly constant across both the non-enhanced and delayed scans to ensure precise measurements and reliable ECV quantification [10,27,57]. Concerning the acquisition timing, the ECV of healthy, remote myocardium remains stable without significant differences at 3, 5, and 7 min [6,17,35]. Therefore, a scan delay between 5 and 7 min serves as a practical compromise for simultaneous ECV and LIE evaluation, guaranteeing reliable measurements and excellent visual scar delineation [1,35], provided that the timing is tailored to the specific disease substrate to optimize overall accuracy. Regarding contrast media usage, the mathematical estimation of ECV via SECT is highly robust as it relies on the tissue-to-blood attenuation ratio rather than absolute enhancement [2,5,17,38]. Recent literature has demonstrated that there is no significant difference in the mean myocardial ECV calculated with standard CCTA doses (0.84 mL/kg at 370 mg I/mL) compared to high-dose protocols (28.7 ± 4.3% vs. 28.7 ± 4.4%, respectively), showing an excellent correlation and minimal bias [38]. However, to address the operational discrepancy with LIE visual requirements and prevent false negatives in scar detection, the use of high-contrast volumes and concentrations remains the recommended standard in comprehensive combined protocols [1,5,17]. Finally, the primary technical challenge of the SECT subtraction method lies in the potential spatial misregistration between the pre- and post-contrast scans. Even minor variations in the patient’s breath-hold depth, diaphragmatic position, or heart rate between the two acquisitions can generate artifacts that compromise the accuracy of the ΔHU calculation [1,6,54]. To mitigate this limitation, the use of advanced post-processing workstations equipped with automatic, three-dimensional non-rigid image registration algorithms is strongly recommended, as they can electronically align the myocardial borders prior to performing the mathematical subtraction [11,16,31,35,38].

## 6. Dosimetry and Radiation Protection

The incorporation of LIE imaging and ECV quantification into standard cardiac SECT protocols may raise primary concerns regarding cumulative radiation exposure [6,10,11]. The subtraction method necessary for calculating SECT-ECV mandates an additional non-enhanced baseline scan, which incrementally increases the total radiation burden and complexity of the workflow [6,16,50]. Consequently, retrospective ECG-gated helical scanning is generally discouraged for the LIE phase, as it can be associated with effective radiation doses ranging from 15 to 21 mSv, depending on scanner generation and protocol optimization, which contradicts the “as low as reasonably achievable” (ALARA) principle [17,45,47,59]. To optimize dosimetry during these acquisitions, performing the delayed enhancement scan using prospective ECG-triggering is recommended [7,9,17]. Several historical studies have demonstrated the feasibility of ultra-low-dose delayed phase acquisitions, albeit with critical clinical caveats. For instance, Bettencourt et al. evaluated patients using a 64-slice SECT scanner with prospective triggering at a fixed tube voltage of 80 kVp, reporting that the delayed enhancement scan was responsible for a comparatively low average radiation exposure of 0.50 ± 0.10 mSv [30]. However, this was achieved with a low tube current setting (160 mAs), without AEC, which significantly compromised image quality in patients weighing over 80 kg due to high image noise [30]. Similarly, Blankstein et al. documented a mean effective dose of 1.2 ± 0.3 mSv for the LIE phase utilizing a prospectively triggered single-energy protocol at 100 kVp [44]. In patients presenting with a low and stable heart rate, the application of a high-pitch helical acquisition mode is suggested to further curtail radiation exposure. In an analysis by Goetti et al., single-energy LIE imaging performed utilizing a high-pitch mode, and 100 kVp (320 mAs, pitch of 3.4) yielded an estimated effective radiation dose of 0.89 ± 0.07 mSv, while maintaining a high per-patient accuracy of 91.7% for detecting myocardial scars [9]. It should be noted that implementing a highly diagnostic low-voltage protocol with a higher reference tube current to suppress noise and avoid photon starvation will result in slightly higher, yet highly competitive, effective doses (typically in the range of 1.5 to 3.0 mSv) [11,26,35]. This clinical benchmark is substantiated by trials utilizing low tube voltages paired with high current settings. For instance, Langer et al. demonstrated that using 80 kVp with an average effective tube current of 614.2 ± 96.4 mAs (up to a maximum of 792 mAs via AEC) yielded high-quality diagnostic images of myocardial fibrosis with an average effective dose of 2.8 ± 0.5 mSv [26]. Similarly, Takaoka et al. reported a delayed-phase effective dose of 2.3 ± 0.1 mSv on a 320-slice scanner using prospective gating at 80 kVp combined with a high tube current of 803 ± 19 mA and model-based iterative reconstruction [42]. Furthermore, an optimized protocol utilizing a 256-slice scanner at an ultra-low tube voltage of 70 kVp paired with DLIR achieved an effective dose of 2.4 ± 0.9 mSv while employing a high average tube current of 1282 ± 70 mA to maintain an excellent contrast-to-noise ratio of 4.3 ± 1.2 [11]. Lastly, Hamdy et al. reported an effective radiation dose of 2.9 ± 0.61 mSv for the combined delayed acquisition of three separate time-points (3, 5, and 7 min) at 80 kVp using a quality reference of 580 mAs, which translates to a highly optimized dose of approximately 1.0 mSv per single late-phase acquisition [35].

## 7. Clinical Applications of LIE and ECV in SECT: Ischemic Heart Disease

### 7.1. Acute Myocardial Infarction (AMI)

In the setting of acute ischemic syndromes and reperfused AMI, SECT may provide morphological parameters of myocardial damage [20,21,60]. Following primary percutaneous coronary intervention (PCI), the evaluation of the myocardium via LIE CT can identify the extent of irreversible necrosis and regions of microvascular obstruction (MVO) [20,21,22]. MVO is characterized by severe capillary damage that prevents the contrast medium from penetrating the necrotic core [14,21,60]. During the LIE phase, the irreversibly damaged but reperfused myocardium demonstrates hyperenhancement, while the central MVO core may persistently appear as a dark, hypoenhanced defect surrounded by the hyperenhanced scar tissue [14,21,46,60]. Data indicate that patients exhibiting myocardial delayed enhancement coupled with MVO on non-contrast multidetector CT acquired immediately post-PCI show a higher likelihood of failed microvascular reperfusion and larger subsequent enzyme release [22,60]. In synthesis, the overall clinical evidence establishes SECT as a potentially rapid-access tool in the acute phase of AMI; by enabling simultaneous assessment of coronary patency, infarct size, and microvascular obstruction (no-reflow), it provides prognostic stratification post-reperfusion and serves as a viable surrogate when MRI is unavailable or contraindicated.

### 7.2. Chronic Ischemic Scar and Myocardial Viability

**Figure 3:**
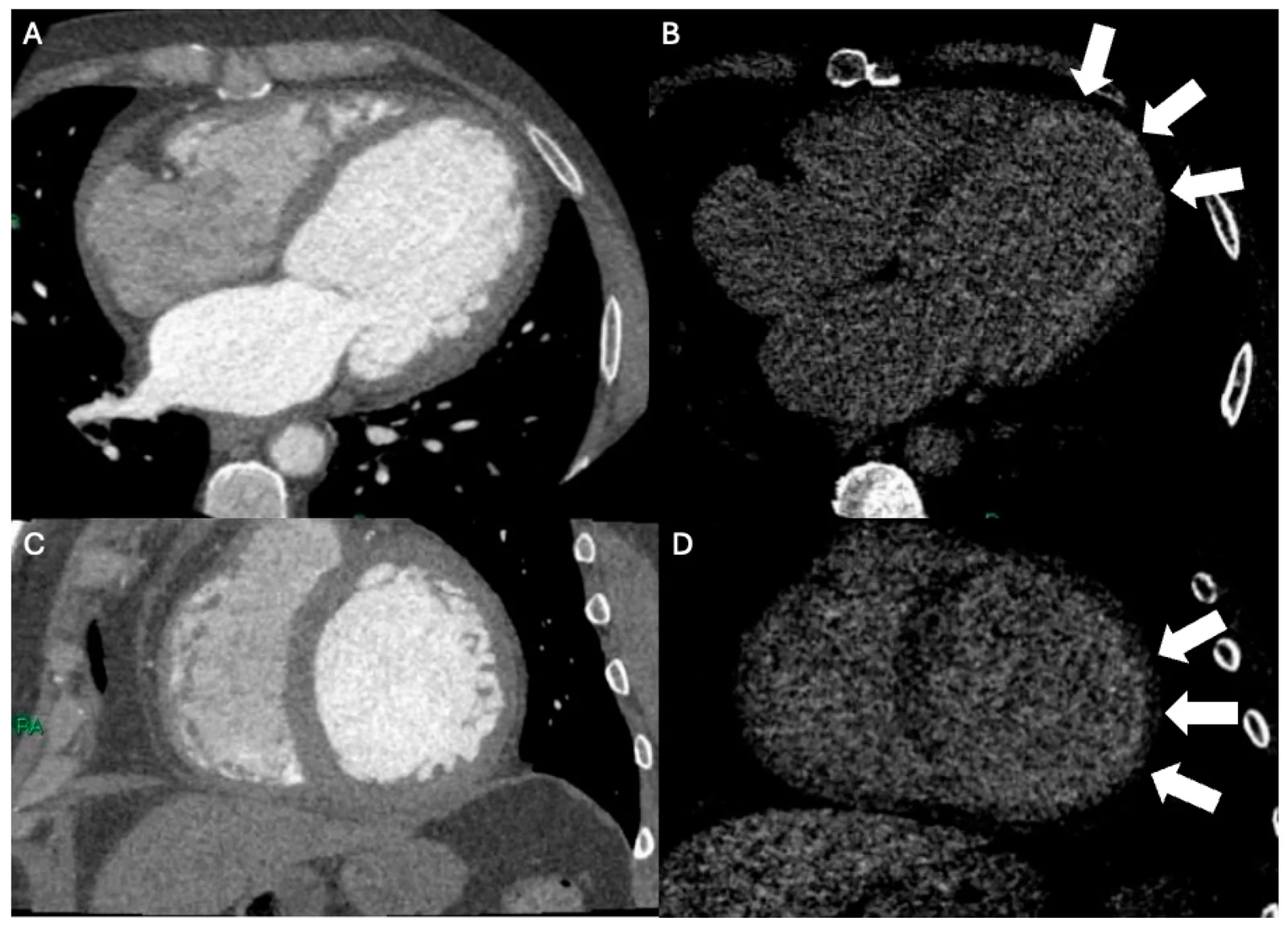
A 65-year-old male with a history of prior myocardial infarction, presenting for pre-procedural myocardial viability assessment due to chronic ischemic heart failure. (**A**,**C**) Arterial phase images (4-chamber and mid-ventricular short-axis) characterized by homogeneous myocardial attenuation, which does not allow for tissue discrimination. (**B**,**D**) Corresponding delayed scans (LIE) reveal a distinct subendocardial hyperdensity at the level of the mid-apical lateral wall, the apex, and the apical septum (arrows), consistent with post-ischemic fibrosis. Side-by-side comparison with the arterial phase delineates the scar borders, overcoming the limitations of tissue iodine density, which tends to merge with the blood pool due to the partial volume effect.

**Figure 4:**
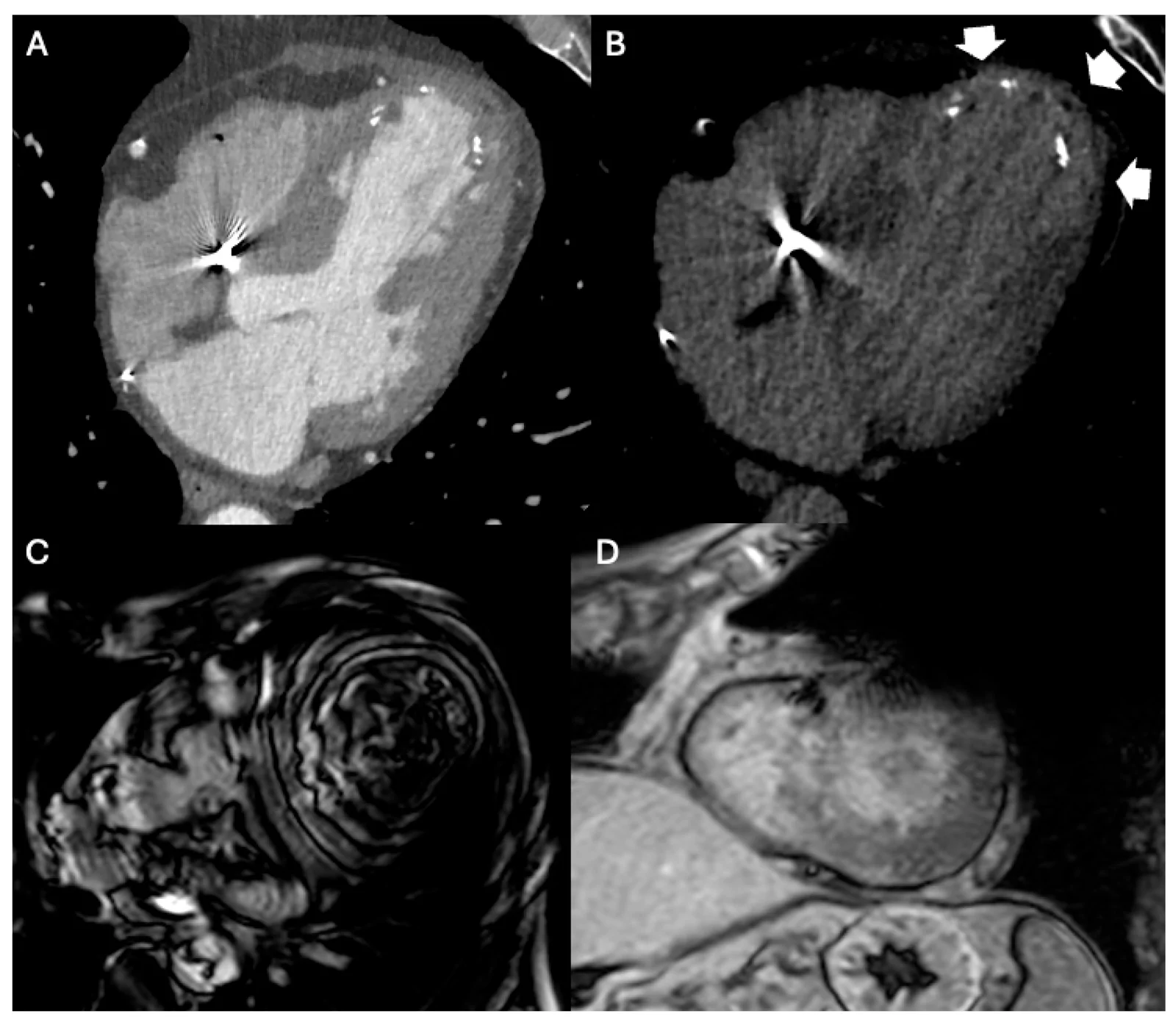
A 72-year-old male with known ischemic cardiomyopathy and an implantable ICD, presenting for myocardial viability assessment due to worsening heart failure. (**A**,**B**) SECT images showing apical and septal transmural Late Iodine Enhancement (LIE) (arrows). (**C**) CMR evaluation in the same patient: diagnostic signal is severely compromised by extensive metallic artifacts originating from the CIED generator, precluding accurate tissue characterization. (**D**) Even the application of wideband LGE sequences results in only partial information recovery.

In patients with chronic coronary artery disease, SECT with LIE has been proposed as a tool to assess myocardial viability prior to surgical or percutaneous revascularization [9,14,20,23,61]. Ischemic scars follow a specific coronary vascular territory and originate from the subendocardium, progressing towards the epicardium depending on the duration of the ischemic insult [1,5,35] (Figure 3). The presence of extensive LIE (with non-viability defined as a scar involving >50% of the myocardial wall thickness) indicates non-viable myocardium with a low probability of functional recovery post-revascularization [9,22,62]. A comparative analysis by Gerber et al. evaluating patients with chronic left ventricular ischemic dysfunction demonstrated that LIE appears to accurately delineate the scar extent, showing a high correlation with magnetic resonance imaging for absolute infarct size [23]. Interestingly, while severe myocardial wall thinning is traditionally considered a definitive sign of irreversible transmural scarring, data indicate that thinned segments may still contain a limited scar burden (≤50% scarring) [63]. In these cases, regional systolic thickening can significantly improve after revascularization [63]. Furthermore, SECT represents an alternative for viability assessment in patients with ischemic cardiomyopathy and implantable cardioverter-defibrillators (ICDs), where CMR evaluation is often non-diagnostic due to extensive metallic artifacts (Figure 4) [2,15].

### 7.3. Prognostic Implications

The quantification of myocardial scar burden using SECT has been shown to hold prognostic relevance [49,60]. In a logistic regression analysis predicting left ventricular remodeling post-AMI, the presence of heterogeneous enhancement was identified as an independent predictor of adverse remodeling, while a separate multivariable Cox proportional hazards analysis showed that the absolute size of LIE was a powerful independent predictor of long-term adverse clinical events [49,60]. Similarly, the identification of extensive scar tissue by CT in patients with ischemic cardiomyopathy correlates with an increased risk of ventricular arrhythmias, heart failure hospitalizations, and major adverse cardiac events [15,49,52,54,57,64,65].

## 8. Clinical Applications of LIE and ECV in SECT: Non-Ischemic Heart Disease

### 8.1. Hypertrophic Cardiomyopathy (HCM)

In hypertrophic cardiomyopathy, the expansion of the extracellular matrix through intramyocardial fibrosis is a hallmark of the disease and serves as a major substrate for ventricular arrhythmias and sudden cardiac death [2,4,24,26]. Scars in HCM typically manifest as patchy, nodular, or streaky enhancement, frequently localizing at the right ventricular insertion points in the basal to mid-interventricular septum or within the most hypertrophied segments [2,3,5,10,53,66]. In a study by Berliner et al. involving 64-slice SECT, the modality detected focal scars with a frequency comparable to that of MRI [24]. The mean focal scar burden quantified by SECT correlated strongly with the measurement detected by MRI, yielding a correlation coefficient (R^2^) of 0.76 [24]. Furthermore, data from Shiozaki et al. indicate that the presence and extent of myocardial fibrosis detected by cardiac CT may hold significant prognostic value, as they are strongly associated with future ventricular fibrillation and tachycardia events, as well as the occurrence of implantable cardioverter-defibrillator firings [3,5,12,64]. ECV expansion in HCM correlates closely with MRI findings and the total mass of focal myocardial fibrosis is directly associated with the risk of fatal ventricular arrhythmias and the frequency of ICD interventions [4,5,64]. In summary, the diagnostic capability of SECT in identifying characteristic mid-wall and junctional fibrosis in HCM matches the spatial distribution of MRI, positioning SECT as a clinical alternative for arrhythmic risk stratification and ICD planning.

### 8.2. Cardiac Amyloidosis and Infiltrative Diseases

Cardiac amyloidosis is an infiltrative disease characterized by the progressive extracellular deposition of misfolded amyloid fibrils, leading to an expansion of the interstitial space [33,48]. On MRI LGE, this extensive expansion presents as a diffuse subendocardial or transmural pattern of hyperenhancement, frequently with a base-to-apex gradient [2,4,5,33]. Given the profound alterations in iodine kinetics caused by the amyloid burden, patients with amyloidosis may present with lower myocardial attenuation during the initial first-pass (angiographic) phase and an anomalous increase in attenuation during the delayed phase, unlike healthy myocardium, where attenuation strictly decreases over time [33]. However, qualitative detection on delayed phase SECT is limited by a low contrast-to-noise ratio, making quantitative CT-ECV assessment or advanced dual-energy/spectral iodine mapping the preferred approaches [27,33]. Beyond qualitative visual assessment, SECT permits the robust quantification of the amyloid burden via the ECV fraction. On CT, this presents as elevated ECV values (often >45–54%) [6,16,18,33]. An elevated ECV is an independent predictor of all-cause mortality, transforming CT from a purely anatomical tool into a powerful prognostic biomarker [6,16,18,52]. In conclusion, SECT serves as a prognostic biomarker, particularly valuable in patients undergoing pre-procedural structural heart evaluations where cardiac amyloidosis frequently co-exists.

### 8.3. Aortic Stenosis and Pre-TAVI/SAVR Planning

Severe aortic stenosis triggers maladaptive myocardial remodeling and diffuse fibrosis [5,16]. A large meta-analysis has established that pre-procedural ECV values > 30.7% are strongly associated with worse cardiovascular outcomes after surgical (SAVR) or transcatheter (TAVI) valve replacement [1,5,52]. Elevated CT-ECV independently predicts heart failure hospitalizations and all-cause mortality, making it a key parameter for risk stratification [5,6,52]. Bandula et al. validated the use of equilibrium contrast-enhanced SECT in patients with severe aortic stenosis, demonstrating that CT-derived ECV significantly correlates with the histological collagen volume fraction from endomyocardial biopsies obtained during surgery, as well as with MRI-derived ECV [25]. It should be explicitly noted that this validation applies strictly to this continuous infusion paradigm and does not directly transfer to the bolus-only delayed subtraction techniques described earlier [25]. Furthermore, evaluating ECV during pre-TAVI planning has emerged as a powerful screening tool for detecting concomitant transthyretin cardiac amyloidosis, which has a reported prevalence of 14–16% in this elderly population [5,16]. Scully et al. demonstrated that global pre-TAVI ECV-CT has an excellent diagnostic accuracy compared to bone scintigraphy, proposing a clinical screening threshold of 31% to prompt confirmatory testing [5,6,67]. In summary, integrating ECV in the TAVI workup provides independent prognostic value.

### 8.4. Acute Myocarditis

In the emergency department, SECT may be a tool for the differential diagnosis of troponin-positive acute chest pain, particularly when coronary CT angiography excludes obstructive coronary artery disease [2,5]. Acute myocarditis typically presents on LIE as patchy or subepicardial or mid-wall layers, typically in lateral/inferolateral walls, thereby sparing the subendocardium [2,4,5,18,54]. In a study by Palmisano et al. analyzing 84 participants with acute chest pain, a comprehensive SECT protocol (including angiography and a 10 min LIE scan) was able to pinpoint the underlying etiology in 90% of cases [18]. Among the 42 patients without obstructive coronary disease, myocarditis was the most frequent diagnosis, identified by SECT in 52% of the cases [18]. The myocardial segments affected by myocarditis also demonstrated a pathologically increased regional ECV (median 30%, interquartile range 28–34%), reflecting acute inflammatory hyperemia, interstitial edema, and cell membrane leakage [18].

### 8.5. Dilated Cardiomyopathy (DCM)

Dilated cardiomyopathy is characterized by progressive ventricular dilation and systolic dysfunction [2,57]. Differentiating non-ischemic DCM from ischemic left ventricular dysfunction is an important clinical step [68]. While ischemic dysfunction is generally characterized by subendocardial or transmural scars mapped to a specific coronary territory, non-ischemic DCM often displays no LIE or presents with a linear, mid-wall septal scar pattern [1,2,5,66,68]. The quantification of myocardial fibrosis using SECT-derived ECV has demonstrated prognostic utility in this population. Yashima et al. reported that in patients with DCM, an ECV value exceeding 32.26% is associated with a significantly higher incidence of major adverse cardiac events [1,5,69]. Similarly, another investigation applying a threshold-based quantification method on CT scans of non-ischemic dilated cardiomyopathy patients found that an optimal myocardial LIE cutoff of 4.3% (using a 4-standard-deviation threshold) was significantly predictive of adverse composite outcomes, including heart failure hospitalization and cardiac death [57]. In non-ischemic dilated cardiomyopathy, the burden of diffuse fibrosis drives the severity of heart failure and arrhythmogenic risk [2,57,66].

### 8.6. Left Ventricular Thrombus (LVT) Detection

In addition to characterizing the myocardium, SECT LIE has demonstrated significant clinical utility in the detection of intracardiac masses, specifically left ventricular thrombi (LVT) [70]. LVT is a high-risk complication of ischemic and non-ischemic cardiomyopathies, associated with systemic thromboembolic events [70]. While transthoracic echocardiography is the standard first-line imaging modality, its sensitivity is often suboptimal, particularly for small or mural thrombi located at the apex [50,70]. Data indicate that incorporating LIE acquisition significantly improves the detection of LVT compared to early-phase (angiographic) scanning. During the LIE phase, the avascular thrombus does not accumulate the contrast medium, creating a stark density difference against the hyperenhanced fibrotic myocardium and the contrast-opacified blood pool [70].

In a study involving 30 patients, receiver operating characteristic (ROC) curve analysis revealed that the area under the curve (AUC) for LVT detection was superior for LIE SECT images (AUC 0.95) and CT-derived ECV maps (AUC 0.98) compared to early-phase images (AUC 0.78) and non-contrast images (AUC 0.55) [42,70]. Furthermore, ECV mapping provided the highest diagnostic performance, yielding a sensitivity of 100% and a specificity of 85.7%, compared to a sensitivity of 92.9% and a specificity of 82.1% for the standard LIE images [70]. Previous quantitative assessments have shown that the mean CT attenuation of LVT is approximately 43.2 ± 15.3 HU, which is significantly lower than that of the surrounding interventricular septum (102.9 ± 23.1 HU), facilitating objective, threshold-based thrombus identification [70]. Because an avascular thrombus does not accumulate contrast media at equilibrium, it shows low ECV values, allowing for clear differentiation between the thrombus, the blood pool, and the underlying infarcted myocardium (which instead shows very high ECV) [70].

### 8.7. Takotsubo Cardiomyopathy

Takotsubo cardiomyopathy (TC) is a complex, often stress-induced cardiomyopathy characterized by transient left ventricular apical ballooning and systolic dysfunction, which can clinically mimic an acute anterior myocardial infarction [2]. Cardiac computed tomography (CT) provides a comprehensive, non-invasive “one-stop shop” tool to evaluate this condition by simultaneously ruling out obstructive coronary artery disease and characterizing the myocardial tissue [2,18]. Mapping the ECV appears to be diagnostic for TC. Patients characteristically exhibit a circumferential increase in ECV in the affected mid-apical segments in the absence of focal scars [2,5,18]. By combining wall motion analysis, the lack of focal LIE, and specific regional ECV elevation, CT effectively distinguishes Takotsubo syndrome from acute myocardial infarction and myocarditis [2,18].

### 8.8. Cardio-Oncology: Early Toxicity Monitoring

An emerging application for CT-ECV is the longitudinal monitoring of myocardial toxicity in patients undergoing cancer therapies (e.g., anthracyclines or thoracic radiotherapy) [5,6,16]. Fibrotic changes and ECV expansion can be quantified in the early stages of tissue damage, acting as an early biomarker long before a measurable decline in left ventricular ejection fraction occurs [5,6,16,50]. Integrating ECV measurements from LIE scans into routine cancer surveillance protocols could facilitate early cardioprotective interventions, though further research is still needed to establish its full clinical impact [16].

### 8.9. Cardiac Sarcoidosis

Cardiac sarcoidosis is a granulomatous disease that can lead to heart blocks and sudden death [2,58]. CT LIE scan accurately detects the patchy, mid-myocardial, or subepicardial fibrotic lesions typical of sarcoidosis [2,58,71]. Studies demonstrate that CT provides high sensitivity (up to 94%) for detecting sarcoidosis lesions, proving to be a suitable, reliable alternative for disease monitoring in patients contraindicated for MRI [2,4,58]. Furthermore, because granulomatous inflammation and subsequent fibrotic remodeling expand the interstitial space, ECV is significantly elevated in the affected myocardial regions [6,7].

### 8.10. Pulmonary Hypertension

In patients with known or suspected pulmonary hypertension, computed tomography offers a valuable non-invasive tool for myocardial tissue characterization, particularly through the quantification of the ECV [6,16]. Studies comparing CT-derived ECV with the reference standard cardiac magnetic resonance have demonstrated a strong correlation between the two modalities in the interventricular septum and the left ventricular free wall, showing correlation coefficients between 0.73 and 0.79 [6]. However, this diagnostic correlation is notably weaker when assessing the thin right ventricular free wall [6]. From a clinical perspective, the targeted assessment of CT-ECV at the anterior right ventricular insertion points is particularly significant [6]. At this specific anatomical location, elevated ECV values exhibit a good correlation with the patient’s mean pulmonary artery pressure [6]. Consequently, localized ECV quantification using CT serves as a potentially promising non-invasive surrogate marker for evaluating the overall disease severity and hemodynamic impairment in pulmonary hypertension [6]. In this clinical scenario, chronic pulmonary hypertension represents the primary driver for right ventricular remodeling and secondary tricuspid regurgitation, where advanced risk stratification is crucial to determine prognosis and avoid futile interventions [72].

A summary of the diverse clinical presentations, specific patterns, and the clinical and prognostic implications of LIE and ECV across both ischemic and non-ischemic conditions is provided in Table 2.

## 9. Technical Challenges and Limitations in Clinical Interpretation

When translating the reported diagnostic performance of SECT-LIE and ECV quantification into standard clinical practice across various cardiac pathologies, the physical boundaries of single-energy systems should be integrated into the interpretation of results. The first major physical limitation is the inherently lower CNR of iodine on CT delayed scans compared to gadolinium on LGE-MRI [2,9,11]. Missed scars on SECT are predominantly of non-ischemic etiology, characterized by a low overall scar burden (<6%) and a lower CNR compared to ischemic scars [3,15]. Similarly, in ischemic heart disease, although SECT demonstrates high specificity for identifying transmural infarctions, its segment-level sensitivity remains suboptimal for the visual detection of small subendocardial scars [5,12,30]. The second physical limitation is the reliance of SECT-ECV on the image-subtraction technique, which requires two separate cardiac phases [1,31,38,55]. Because these scans are acquired several minutes apart, any variation in breath-holding depth, diaphragmatic position, or chest motion introduces spatial misregistration. In patients with advanced heart failure or dilated cardiomyopathy, who often suffer from orthopnea or dyspnea, the inability to maintain consistent breath-holds can generate motion artifacts and misalignment during automatic non-rigid image subtraction. Furthermore, subtraction-based SECT-ECV is sensitive to irregular heart rates and irregular cardiac gating [1,31,54]. For example, in cardiac amyloidosis, where diffuse amyloid deposition expansion can globally involve the myocardium, there is a progressive accumulation of contrast agent within the extracellular matrix [4,5,27,33]. This accumulation results in a marked reduction in the contrast-to-noise ratio between the blood pool and the left ventricular myocardium [6,33]. Consequently, visual qualitative analysis can be inaccurate (yielding 15% false negatives and 27% false positives), making quantitative ECV recommended [4,33]. However, the high prevalence of atrial fibrillation in cardiac amyloidosis patients (often exceeding 30% to 40%) can compromise cardiac gating, producing spatial misregistration artifacts and significant segmental variability in subtraction-derived ECV maps [27,48]. Furthermore, myocarditis frequently presents with chest pain and palpitations, whereas cardiac sarcoidosis typically manifests with conduction abnormalities, ventricular arrhythmias, or heart failure symptoms; irregular cardiac cycles and breath-hold instabilities severely degrade subtraction co-registration [2,5,54].

## 10. The Influence of Reader Experience on Diagnostic Performance

A study by Palmisano et al. evaluated the impact of reader experience on the diagnostic performance of LIE-CT by comparing the results of two readers (with 8 years and 2 years of experience, respectively) against LGE-MRI as the reference standard in 40 consecutive patients. The analysis demonstrated that the scar burden estimated by LIE-CT correlated more strongly with MRI for the highly experienced reader than for the less experienced reader [3]. In a per-patient analysis, the experienced reader achieved an accuracy of 95% and a sensitivity of 93%, whereas the less experienced reader achieved an accuracy of 88% and a sensitivity of 83% [3]. Notably, specificity remained excellent across both readers (100% per-patient and 99% per-segment) [3]. The study further highlighted that the scars missed by the readers were predominantly of non-ischemic etiology, characterized by a low overall scar burden (<6%) and a significantly lower contrast-to-noise ratio compared to ischemic scars [3]. These findings indicate that while LIE-CT appears to be a reliable method for identifying ischemic scars, the detection of subtle, non-ischemic subepicardial or mid-wall lesions requires specific expertise; therefore, dedicated training is advised to maximize the diagnostic yield of the modality [3].

### Practical Implications and Implementation in Centers with Limited Experience

The discrepant diagnostic performances between experienced and inexperienced readers carry significant practical implications for centers seeking to adopt single-energy late iodine enhancement in clinical routine [3]. However, since both highly experienced and less experienced readers demonstrate excellent specificity and excellent positive predictive value, a positive finding of delayed enhancement on LIE-CT can be trusted with good diagnostic confidence. This indicates that positive LIE-CT findings are highly reliable for confirming the presence of myocardial scars, regardless of the reader’s level of experience [3]. Conversely, negative scans evaluated by less experienced readers must be interpreted with caution. Novice readers show a significant deficit in sensitivity compared to experts, both at the patient level (83% vs. 93%) and particularly at the segment level (62% vs. 85%) [3]. Underdiagnosis primarily occurs with small, thin, subepicardial or mesocardial non-ischemic scars that typically present with a low overall scar burden and a lower CNR compared to ischemic scars [3]. Therefore, a negative visual scan cannot be considered completely reliable to exclude the presence of subtle non-ischemic substrates, and several clinical strategies could be implemented in low-experience centers to protect patient safety:

1. Expert Review of Negative Scans: Because the negative predictive value is compromised for less experienced operators (69% vs. 85% at the patient level), any negative LIE-CT scan should be reviewed by a highly experienced reader [3].
2. Dedicated Cardiological and Radiological Training: Centers initiating a LIE-CT service should establish dedicated cardiovascular imaging training to help readers overcome the steep learning curve associated with identifying subtle lesions.
3. Prioritization of Quantitative ECV Mapping: Visual evaluation of delayed-contrast scans is inherently subjective and challenging for inexperienced readers. Centers should utilize quantitative, color-coded ECV maps. The introduction of color-coded parametric mapping provides objective, quantitative tissue characterization, enabling even inexperienced individuals to easily perform the diagnostic evaluation [3,11].
4. Adoption of Advanced Reconstructions and Denoising Filters: Reconstructing scans with advanced algorithms can suppress quantum noise, increase the CNR, and improve sensitivity for detecting myocardial lesions [13,19,40]. Furthermore, post hoc deep learning denoising filters can reduce image noise, enhance per-patient sensitivity, and improve overall diagnostic accuracy [13,19,40].

## 11. Comparison of Diagnostic Accuracy Between Technologies

Aggregated data from the recent meta-analysis by Gatti et al. (2025) offer a comprehensive perspective on the diagnostic accuracy of computed tomography for myocardial tissue characterization [12]. Although the study includes evaluations performed with both DECT and SECT technologies, the majority of the 14 analyzed clinical studies (involving 526 patients and 5758 myocardial segments) are based on the use of SECT scanners [12]. Crucially, these diagnostic values represent overall, all-CT pooled estimates that combine both SECT and DECT technologies. Specifically, at the patient level, LIE CT exhibits a pooled sensitivity of 0.96 and a specificity of 0.95 [12]. This high sensitivity may establish a highly effective diagnostic “rule-out” profile at the patient level, allowing clinicians to potentially exclude the presence of myocardial fibrosis. In contrast, at the segment level, where the overall pooled sensitivity is 0.86 and specificity reaches 0.98, the modality may serve as a highly specific “rule-in” tool, optimal for confirming and localizing transmural or moderate-to-large subendocardial scars [12]. Nonetheless, the detection of subtle or millimetric subendocardial lesions remains a challenge for single-energy systems, as SECT segment-level sensitivity is significantly lower at 83% compared to the 93% achieved by DECT, which benefits from spectral monoenergetic reconstructions and iodine-density maps [12]. Therefore, while SECT-driven segment-level evaluations provide high specificity, CMR remains the gold standard for high-contrast, high-resolution myocardial tissue characterization [3,5,12,23]. Similarly, for ECV quantification, DECT may offer advantages as it relies on direct iodine mapping rather than the subtraction method, thereby avoiding misregistration artifacts caused by motion or irregular heart rates [1,6,17]. This technological advantage is supported by meta-analyses from Han et al. and Zhang et al., which revealed that ECV derived from DECT iodine-specific images exhibits a significantly higher pooled correlation with CMR than the SECT subtraction method [17,73,74]. Clinical studies further validate this trend; Tavoosi et al. evaluated patients with cardiac amyloidosis and found that DECT-derived global ECV had a stronger correlation and higher intraclass correlation coefficient with CMR compared to SECT [27]. The recent introduction of PCD-CT has further elevated diagnostic capabilities by offering inherent spectral separation with exceptionally high spatial and temporal resolution [17]. Popp et al. confirmed that spectral reconstruction derived from PCD-CT provides slightly stronger correlations with CMR [48]. Ultimately, while SECT provides acceptable baseline accuracy, DECT and PCD-CT technologies offer significantly superior and more reliable diagnostic performance for both LIE detection and ECV quantification.

## 12. Methodological Considerations and Limitations

Several limitations of both the available literature on SECT myocardial characterization and this review must be acknowledged. First, the methodological design as a narrative synthesis precludes a quantitative meta-analysis of diagnostic thresholds and pooled accuracy metrics, inherently carrying a degree of selection bias despite our purposive literature search. Second, the majority of clinical studies evaluating single-energy LIE and ECV are characterized by retrospective, single-center designs with relatively small patient cohorts, which may limit the generalizability of their findings. Third, the included literature exhibits significant heterogeneity regarding SECT acquisition protocols, contrast media administration strategies, and the specific CT scanner generations utilized, which limits direct comparability of the results. Fourth, most validation studies have relied on LGE or T1 mapping by CMR as the imaging reference standard rather than direct histopathological validation from myocardial biopsy specimens.

## 13. Conclusions

In conclusion, SECT represents a highly accessible and valuable alternative to CMR for non-invasive myocardial tissue characterization in both ischemic and non-ischemic cardiomyopathies. By enabling the qualitative assessment of LIE and the quantitative estimation of ECV, SECT extends the clinical utility of cardiac CT beyond conventional coronary anatomy. However, the routine integration of SECT remains fundamentally bounded by physical and technical limitations. To maximize diagnostic performance, systematic protocol optimization is essential. Furthermore, because qualitative image interpretation is strongly dependent on reader experience, centers adopting this modality should prioritize dedicated training programs and implement color-coded quantitative ECV mapping to minimize false-negative findings and ensure reproducible diagnostic evaluations. While spectral imaging modalities, such as DECT and PCD-CT, represent the technological future of the field, optimizing existing SECT protocols remains highly clinically relevant for maximizing the potential of the widely installed scanner base. Ultimately, while SECT offers acceptable baseline diagnostic accuracy, it should be utilized as a robust, complementary clinical resource rather than a complete replacement for the reference-standard CMR.

## Figures and Tables

**Table 1:** SECT acquisition parameters for late iodine enhancement (LIE).

Parameter Category	Recommendation and Details
Contrast Media Concentration	Use a high-concentration contrast agent (e.g., 350–400 mg I/mL) to increase overall attenuation [1,5,24,26,27,28].
Contrast Volume/Dose	Tailor contrast volume to the patient’s body weight. A weight-based volume of 1.4 to 1.8 mL/kg of high-concentration agent is recommended as a pragmatic clinical standard to ensure acceptable lesion delineation [1,6,10,28,29,30].
Injection Strategy	A split-bolus or triphasic injection approach is commonly reported to achieve interstitial accumulation without compromising the initial angiographic phase [3,6,11,14,15,27,28].
Acquisition Timing (Delay Time)	Thus, while a 5–10 min range serves as a general guide, the scan delay should be tailored to the specific pathological target based on contrast kinetics. For example, the literature suggests 5 min for acute, reperfused myocardial infarction or cardiac amyloidosis; 5 to 7 min for chronic myocardial scars; 10 min for acute inflammatory states (active myocarditis); 7 min for hypertrophic cardiomyopathy; 6 to 10 min for non-ischemic dilated cardiomyopathy [1,2,3,6,10,11,14,15,20,21,26,31,32,33,34,35].
X-ray Tube Voltage (kVp)	Reducing tube voltage to 80 kVp is recommended to significantly increase iodine attenuation by shifting the X-ray spectrum closer to the iodine K-edge [3,5,36,37]. For obese patients, a moderate increase to 100 kVp is suggested to prevent photon starvation while maintaining contrast [1,3,4,14,30,36].
X-ray Tube Current (mAs)	To counteract the increased image noise from lower tube voltages, it is recommended to employ automatic exposure control (AEC) systems with a high reference tube current-time product (e.g., 500–600 mAs) [11,13,22,26,35,36,37,38,39].
Reconstruction Algorithms	Advanced reconstruction algorithms, including Hybrid Iterative Reconstruction, Knowledge-based Iterative Model Reconstruction (IMR), and Deep Learning Image Reconstruction (DLIR), are recommended to improve image quality and diagnostic performance [11,36,40,41,42].
Additional Noise Reduction	Image noise can be reduced through spatial frequency filtration and image averaging by acquiring and co-registering multiple consecutive heartbeats [13,35,38,43]].
Slice Thickness	Adopt a hybrid reconstruction and visualization strategy. Reconstruct axial source images with a thin slice thickness (typically 1.0 to 1.5 mm, or 0.75 mm) to preserve spatial resolution, minimize partial-volume effects, and allow accurate multiplanar reformations [10,17,24,39]. For rapid visual screening, thicker short-axis reformations (e.g., 5 to 10 mm in average intensity projection) are utilized, but they should always be cross-referenced with thin-slice datasets to prevent missing subtle subendocardial lesions [2,8,10,14,30,35,44,45].
ECG Gating and Scanning Mode	Prospective ECG-triggering is recommended to optimize dosimetry. In patients with a low and stable heart rate, a high-pitch helical acquisition mode is suggested to further curtail radiation [7,9,15,41,46,47].

**Table 2:** Clinical applications and prognostic value of LIE and ECV via single-energy CT in ischemic and non-ischemic heart diseases.

Condition	Tomographic Findings (LIE/ECV)	Clinical and Prognostic Implications
Acute Myocardial Infarction (AMI)	Reperfused myocardium demonstrates hyperenhancement, whereas MVO presents as a central hypoenhanced defect surrounded by the scar [16,23,47,62].	The presence of late iodine enhancement coupled with MVO indicates a higher likelihood of failed microvascular reperfusion and larger subsequent enzyme release [24,62].
Chronic Ischemic Scar and Viability	Ischemic scars map to specific coronary territories and progress from the subendocardium towards the epicardium; a scar extent >50% of wall thickness indicates non-viability [1,4,37].	Useful for assessing viability prior to revascularization; severely thinned segments may still exhibit functional recovery if the scar burden is limited (≤50%) [25,65].
Hypertrophic Cardiomyopathy (HCM)	Intramyocardial fibrosis manifests as patchy, nodular, or streaky enhancement, frequently localizing at right ventricular insertion points and hypertrophied segments [2,4,9,12,26,50].	ECV expansion correlates closely with MRI findings and focal scar burden are directly associated with the risk of fatal ventricular arrhythmias and ICD interventions [4,11,66].
Cardiac Amyloidosis	Qualitative visual detection is limited by low contrast-to-noise ratio. Markedly elevated ECV values (>45–54%) [2,3,4,6,11,18,35]	An elevated ECV acts as an independent prognostic biomarker that powerfully predicts all-cause mortality [3,6,18,54]
Aortic Stenosis	Severe aortic stenosis triggers diffuse fibrosis. Pre-procedural ECV values > 30.7% are strongly associated with worse cardiovascular outcomes after surgical or transcatheter valve replacement [1,4,6,52,54]	Independently predicts heart failure hospitalizations and mortality post-valve replacement; additionally serves as a screening tool for concomitant transthyretin cardiac amyloidosis [4,6,54].
Acute Myocarditis	Patchy, subepicardial, or mid-wall hyperenhancement (typically inferolateral) sparing the subendocardium, along with pathologically increased regional ECV [2,3,4,11,56].	Enables the differential diagnosis of troponin-positive acute chest pain in the emergency department by identifying inflammatory edema without obstructive coronary disease [2,4].
Dilated Cardiomyopathy (DCM)	Frequently lacks LIE or displays a linear, mid-wall septal scar pattern, combined with an elevated ECV or LIE burden [1,2,4,26,69,70]	Differentiates non-ischemic from ischemic left ventricular dysfunction; diffuse fibrosis burden predicts major adverse cardiac events, heart failure severity and cardiac death [1,4,69,70].
Left Ventricular Thrombus (LVT)	LVT is avascular and exhibits low ECV, differentiating it from hyperenhanced fibrotic myocardium and the blood pool [71].	Improves diagnostic performance over early-phase CT and echocardiography for identifying systemic thromboembolic sources [71].
Takotsubo Cardiomyopathy	Characterized by a circumferential regional increase in ECV within the affected mid-apical segments, in the absence of focal scars [2,3,4].	Distinguishes Takotsubo syndrome from acute anterior myocardial infarction or acute myocarditis through a non-invasive, “one-stop shop” assessment [2,3].
Cardio-Oncology	Early fibrotic changes resulting in measurable ECV expansion [4,6,18,52].	Acts as a longitudinal, early biomarker for myocardial toxicity preceding a measurable decline in left ventricular ejection fraction [4,6,18,52].
Cardiac Sarcoidosis	Patchy mid-myocardial or subepicardial fibrotic lesions with significantly elevated regional ECV driven by granulomatous inflammation [6,7,11,64,72].	Provides high sensitivity (up to 94%) for detecting fibrotic lesions, serving as a reliable monitoring alternative for patients with MRI contraindications [2,11,60].
Pulmonary Hypertension	Localized elevation of ECV at the anterior right ventricular insertion points [6].	Serves as a potentially promising non-invasive surrogate marker to evaluate overall disease severity and hemodynamic impairment (mean pulmonary artery pressure) [6].

## Data Availability

No new data were created or analyzed in this study. Data sharing is not applicable to this article.

## Abbreviations

The following abbreviations are used in this manuscript:

AEC	Automatic Exposure Control
ALARA	As Low As Reasonably Achievable
AMI	Acute Myocardial Infarction
AUC	Area Under the Curve
BMI	Body Mass Index
CCTA	Coronary Computed Tomography Angiography
CMR	Cardiac Magnetic Resonance
CNR	Contrast-to-Noise Ratio
CT	Computed Tomography
DCM	Dilated Cardiomyopathy
DECT	Dual-Energy Computed Tomography
DLIR	Deep Learning Image Reconstruction
ECG	Electrocardiogram
ECV	Extracellular Volume
FBP	Filtered-Back Projection
Gd	Gadolinium
HCM	Hypertrophic Cardiomyopathy
HU	Hounsfield Unit
I	Iodine
ICD	Implantable Cardioverter-Defibrillator
IMR	Iterative Model Reconstruction
IR	Iterative Reconstruction
kVp	X-ray Tube Voltage
LGE	Late Gadolinium Enhancement
LIE	Late Iodine Enhancement
LVT	Left Ventricular Thrombus
MRI	Magnetic Resonance Imaging
mAs	X-ray Tube Current
MVO	Microvascular Obstruction
PCD-CT	Photon-Counting Detector Computed Tomography
PCI	Percutaneous Coronary Intervention
ROC	Receiver Operating Characteristic
ROI	Regions of Interest
SAVR	Surgical Aortic Valve Replacement
SECT	Single-Energy Computed Tomography
SNR	Signal-to-Noise Ratio
TAVI	Transcatheter Aortic Valve Replacement
TC	Takotsubo Cardiomyopathy
